# Rosiglitazone Inhibits Adrenocortical Cancer Cell Proliferation by Interfering with the IGF-IR Intracellular Signaling

**DOI:** 10.1155/2008/904041

**Published:** 2008-07-28

**Authors:** Giulia Cantini, Adriana Lombardi, Elisabetta Piscitelli, Giada Poli, Elisabetta Ceni, Sara Marchiani, Tonino Ercolino, Andrea Galli, Mario Serio, Massimo Mannelli, Michaela Luconi

**Affiliations:** ^1^DENOthe Center of Excellence for Research, Transfer and High Education: Section of Endocrinology, Department of Clinical Physiopathology, University of Florence, Viale Pieraccini 6, 50139 Firenze, Italy; ^2^DENOthe Center of Excellence for Research, Transfer and High Education: Section of Gastroenterology, Department of Clinical Physiopathology, University of Florence, Viale Pieraccini 6, 50139 Firenze, Italy; ^3^DENOthe Center of Excellence for Research, Transfer and High Education: Section of Andrology, Department of Clinical Physiopathology, University of Florence, Viale Pieraccini 6, 50139 Firenze, Italy

## Abstract

Rosiglitazone (RGZ), a thiazolidinedione ligand of the peroxisome proliferator-activated receptor (PPAR)-*γ*, has been recently described as possessing antitumoral properties. We investigated RGZ effect on cell proliferation in two cell line models (SW13 and H295R) of human adrenocortical carcinoma (ACC) and its interaction with the signaling pathways of the activated IGF-I receptor (IGF-IR). We demonstrate a high expression of IGF-IR in the two cell lines and in ACC. Cell proliferation is stimulated by IGF-I in a dose- and time-dependent manner and is inhibited by RGZ. The analysis of the main intracellular signaling pathways downstream of the activated IGF-IR, phosphatidyl inositol 3-kinase (PI3K)-Akt, and extracellular signal-regulated kinase (ERK1/2) cascades reveals that RGZ rapidly interferes with the Akt and ERK1/2 phosphorylation/activation which mediates IGF-I stimulated proliferation. In conclusion, our results suggest that RGZ exerts an inhibitory effect on human ACC cell proliferation by interfering with the PI3K/Akt and ERK1/2 signaling pathways downstream of the activated IGF-IR.

## 1. INTRODUCTION

Adrenocortical carcinoma (ACC) is a rare tumor with an incidence of approximately 1–2 per million population per year. ACC is a very aggressive tumor, characterized by a poor prognosis: approximately 50% of patients do not survive beyond 2 years from the diagnosis and the 5-year mortality rate is between 20% and 60% [1]. Its poor prognosis depends mainly upon the limited therapeutic resources. At present, a complete surgical removal following an early diagnosis is the only valuable option for the tumor's cure. Moreover, other than improved surgical management, the prognosis for ACC has not changed significantly over the past three decades [2]. The tumor is, in fact, resistant to radio- and chemotherapy and medical treatment very rarely leads to a complete remission in the case of recurrences or metastatic spread. Although several new medical therapeutic options have been recently proposed [3], at present the medical treatment of advanced ACC is far from being satisfactory, due to our poor knowledge of the molecular mechanisms leading to malignant transformation of adrenocortical cells. In fact, although some intracellular signaling pathways have been shown to be altered in ACC cells [4], efforts to identify the events leading to neoplastic transformation and tumor invasiveness have met with limited success. The role of IGF-I system in mediating proliferation and progression has been well documented in several cancers, including adrenocortical carcinoma [5]. In particular, ACC, as well as the H295R cell line [6], overexpress both IGF-II [7] and its promiscuous receptor IGF-IR [8] in comparison to adrenal adenomas and normal adrenal tissue. The overexpressed IGF-II is thought to act in a paracrine fashion through the IGF-IR to sustain tumor and cell proliferation [6, 9, 10].

Peroxisome proliferator activated receptor (PPAR)-*γ* is a ligand-activated transcriptor factor and a member of the nuclear hormone receptors superfamily. Thiazolidinediones (TZDs), which are a family of PPAR-*γ* ligands, have been introduced in the therapy of type 2 diabetes mellitus (T2D) because of their ability to reduce insulin resistance. In the last few years, an increasing amount of experimental data showing the ability of these drugs to exert additional pleiotropic actions such as regulation of inflammatory processes and of cancer cell growth has been published [11].

TZD effects seem to be mainly due to their ability to bind and activate PPAR*γ* differentially expressed in adipose and other tissues. Upon ligand binding, PPAR*γ* heterodimerizes with the 9-cis retinoic acid receptor (RXR) on specific responsive elements in the promoters of genes involved in glucose and insulin homeostasis, lipid metabolism, and cellular differentiation. Besides this transactivating activity, a ligand-dependent transcriptional transrepression mechanism involving PPAR*γ*/RXR complex has been described. According to such a model, the heterodimerized receptor represses gene transcription in a DNA-binding independent way by physically sequestering activated transcriptional factors or their coactivators [12]. More recently, an increasing number of TZD effects, and in particular the antineoplastic ones, have been shown to be independent of PPAR*γ* activity [13]. Finally, rapid nongenomic activities of TZDs, not resulting in modulation of gene transcription but affecting posttranslational modifications involved in cell signaling, have been reported [14].

In addition to their action as insulin sensitizers, TZDs inhibit cell growth in breast, colon, prostate, lung, pancreas, stomach, thyroid, liver and adrenal cancers [15]. However, the molecular mechanisms underlying such pharmacological activities remain to be elucidated.

Rosiglitazone (RGZ) and pioglitazone (PGZ), the two most widely used PPAR*γ* agonists, have been shown to inhibit growth and invasiveness of the human adrenal cancer cell line H295R [16] as well as to induce cell differentiation and apoptosis [10]. Moreover, H295R cells and both normal and tumoral adrenal tissue express PPAR*γ*, with no differences in the level of expression between tumoral and normal tissue and no correlation with clinical parameters such as tumor size, hormonal profile, and so forth [10, 16].

In this study, we investigate two different cell models of ACC, namely, SW13 and H295R lines, whether the TZD RGZ may exert its antiproliferative action on human ACC cell lines by interfering with the intracellular pathways activated by IGF-IR.

## 2. MATERIALS AND METHODS

### 2.1. Reagents

Anti-phospho [Akt (Ser473), ERK1/2 (T202/Y204)] and anti-Akt antibodies were from Cell Signaling Technology, Inc. (Danvers, Mass, USA). Anti-ERK1/2, anti-IGF-IR*β*, and anti-actin antibodies were from Santa Cruz Biotechnology, Inc. (Santa Cruz, Calif, USA). Anti-PI3K p85 regulatory subunit antibody was from Upstate Biotechnology (Lake Placid, NY, USA). Rosiglitazone was from Alexis Biochemicals (San Diego, Calif, USA). MTS solution was purchased from Promega (Madison, Wis, USA). IGF-I was from Sigma Aldrich (San Louis, Mo, USA). [Methyl-^3^H]thymidine ([^3^H]TdR) was purchased from NEN Life Science Products (Boston, Mass, USA). NVP-AEW541 was provided by Novartis (Basel, Switzerland).

### 2.2. Tissue specimens and cell cultures

A total of three normal human adrenal glands, three adrenal carcinomas, and three adenomas were used in this study. Normal adrenal glands were removed during an expanded nephrectomy due to renal carcinoma or from organ donors (age 32–72 years). Approval for the use of human material was given by the Local Ethical Committee. Informed consent was obtained from each patient. Adrenocortical fragments, collected immediately after surgery, were snap frozen in liquid nitrogen and stored at −80°C.

The human ACC cell lines H295R and SW13 were obtained from the American Type Culture Collection (Manassas, Va, USA). SW13 were cultured in DMEM/F-12 medium (Sigma-Aldrich) with 10% FBS (Euroclone), 2 mM L-glutamine, 100 U/ml penicillin, and 100 *μ*g/ml streptomycin. H295R need DMEM/F-12 medium enriched with a mixture of insulin/transferrin/selenium (Sigma-Aldrich). Cells were incubated at 37°C in a humidified 5% CO_2_ atmosphere.

Subconfluent cells starved 24 hours were treated with different stimuli added to serum-free medium. Rosiglitazone was added simultaneously with IGF-I, at the doses and for the time intervals (15 minutes for western blot analysis and from 24 hours up to 7 days for proliferation experiments) indicated in figure legends.

Cells were pretreated 1 hour with NVP-AEW541 before addition of other stimuli. For incubations longer than 24 hours, media and stimuli were replaced every day.

### 2.3. MTS assay

SW13 and H295R were seeded in 96-well plates at the density of 3·10^3^ and 8·10^3^ cells/well, respectively. After 24-hour starvation in serum-free (SF) medium, cells were treated with the different stimuli in SF medium for the indicated times (see figure legends) and cell number in each well was evaluated by MTS assay (Promega), according to the manufacturer's instructions. The samples were analyzed by an ELISA plate reader (Wallac 1420 - PerkinElmer) at 490 nm wavelength to measure optical density (OD). Each experimental point was performed in quintuplicate in at least three independent experiments.

### 2.4. Cell proliferation assays

#### 2.4.1. Viable cell counting

Cells were seeded in 12-well plates (1,8·10^4^ and 5·10^4^ cells/well for SW13 and H295R, resp.), and after 24-hour starvation were treated for 2 or 4 days in SF medium, then trypsinizedand counted in the haemocytometer. Mean cell number was obtained by counting triplicate in three different experiments. Dead cells were excluded by trypan blue exclusion test.

#### 2.4.2. DNA synthesis assay: [^3^H]thymidine uptake

DNA synthesis was evaluated according to the amount of [^3^H]TdR incorporated into trichloroacetic acid (TCA)-precipitated materials. Cells seeded at different density (5·10^4^ or 2,5·10^4^ H295R cells/well in 12 or 24 well plates, and 1,8·10^4^ SW13 cells/well in 12 well plates) were grown in 10% FBS complete medium till 70% confluence. After 24-hour starvation, cells were treated with IGF-I or RGZ in 1% FBS medium for the indicated times (1–7 days) pulsing them with 1.0 *μ*Ci/ml [^3^H]TdR (6.7 Ci/mmol) 4 hours before stopping proliferation in ice-cold 10% TCA. After washing in TCA and then in methanol, cells were solubilized in 0.2 N NaOH, and radioactivity was measured in the scintillation counter. Experiments were performed in triplicate and repeated at least three times.

### 2.5. Western blot analysis

Treated cells were extracted in lysis buffer (20 mM Tris, pH 7.4, 150 mM NaCl, 0.5% Triton X-100, 1 mM Na_3_VO_4_, 1 mM PMSF). Thirty *μ*g of proteins measured by Coomassie reagent (BIO-RAD Labs, Hercules, Calif, USA) were loaded onto 8–10% reducing SDS-PAGE. After separation, proteins transferred to nitrocellulose membranes were 1 hour blocked at room temperature in 5% skimmed milk in TTBS (0.1% Tween-20, 20 mM Tris, 150 mM NaCl) and incubated overnight with primary antibodies at appropriate dilutions followed by peroxidase-secondary IgG (1:3000). Proteins were revealed by BM-enhanced chemiluminescence system (Roche Diagnostics, Milan, Italy). Image acquisition and densitometric analysis were performed with Quantity One software on a ChemiDoc XRS instrument (BIO-RAD Labs, Hercules, Calif, USA). All western blots were repeated in at least 3 independent experiments. Membrane re-probing was performed after stripping procedure (Pierce, Rockford, Il, USA).

**PI3 kinase assay**

Treated cells were extracted in lysis buffer A (20 mM Tris, pH 7.4, 137 mM NaCl, 1 mM CaCl_2_, 1 mM MgCl_2_, 1% NP-40, 1 mM Na_3_VO_4_, 1 mM PMSF). After protein measurement, aliquots containing equal amount of proteins (300 *μ*g) were precleared with 50 *μ*l of Protein G-Sepharose. Precleared lysates were then immunoprecipitated overnight at 4°C with 3 *μ*g of rabbit anti-p85 PI3K antibody in the presence of 50 *μ*l of Protein A-Sepharose. Sepharose beads washed in a 10 mM Tris-HCl (pH 7.4) containing 0.1 mM EGTA and 5 mM LiCl, were suspended in a kinase buffer (10 mM Tris-HCl, 150 mM NaCl, 5 mM EDTA) containing 20 *μ*g of L-*α*-phosphatidyl inositol (Sigma-Aldrich, St. Louis, Mo, USA), 25 mM MgCl_2_ and 10 *μ*Ci of [*γ* ^32^P]ATP and incubated for 20 minutes at room temperature. The reaction was stopped by the addition of 60 *μ*l of 6 M HCl + 160 *μ*l of chloroform:methanol (1:1). Lipids were then resolved by thin layer chromatography plates (TLC silica gel 60) (Merck Laborchimica, Florence, Italy) in chloroform, methanol, water and ammonium hydroxide (60:47:11, 3:2). Dried TLC sheets were developed by autoradiography. Band quantification was performed with ChemiDoc XRS instrument (BIO-RAD Labs, Hercules, Calif, USA).

### 2.6. Statistical analysis

Results are expressed as mean ± SE. The effect of different concentrations of IGF-I and RGZ on cell proliferation was tested by One-Way ANOVA. Multiple *Post Hoc* comparisons were performed by Bonferroni's correction. Student's *τ*-test for paired or unpaired data was applied when appropriate for comparison of two sets of data. *P* < .05 was taken as significant.

## 3. RESULTS

**Figure 1:**
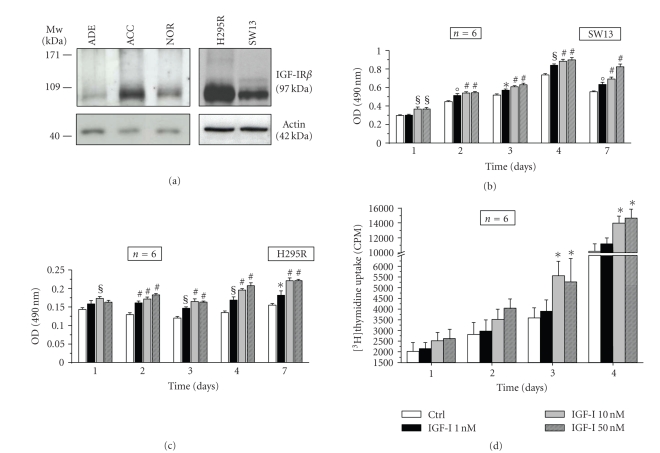
*Adrenocortical cancer is responsive to IGFs*. (a) Western blot analysis of adrenocortical tissues (adrenocortical carcinoma: ACC; normal adrenal tissue: NOR; adrenocortical adenoma: ADE) and adrenocortical cancer cell lines (SW13 and H295R) reveals a high expression of IGF-IR in cancer tissue and cells and lower levels in normal and adenoma tissues. Western blot for actin was performed for lane protein normalization. Molecular weight (Mw) markers are indicated. The effects of cell incubation with increasing doses of IGF-I (1, 10 and 50 nM) for the indicated times on cell proliferation were evaluated by MTS in SW13 (b) and H295R (c) and confirmed in H295R by evaluation of [^3^H]TdR incorporation in cell DNA content (d). Data represent mean ± SE OD (b), (c) or mean ± SE of [^3^H]TdR incorporation (counts per minute, cpm) (d). Statistical significance versus respective control:*P < .05,°*P* < .01, §*P* < .005, #*P* < .001.

Adrenal cancer is characterized by an increased expression of the IGF-IR compared to both nontumoral and adenomal adrenal tissues as demonstrated by western blot analysis with a specific antibody against the *β* subunit of IGF-IR (Figure 1(a)). A marked expression of this receptor is also present in SW13 and in particular in H295R adrenocortical cancer cells (Figure 1(a)), suggesting that both tissue and cell systems may be highly responsive to the effects of IGF-I and IGF-II.

In order to study the role of IGF-IR and of its downstream intracellular signaling pathways on cell proliferation, we stimulated both SW13 and H295R cells with increasing concentrations of the receptor's elective ligand, IGF-I. IGF-I is able to induce cell proliferation in a time- and dose-dependent manner as demonstrated by evaluating cell viability through MTS assay in both SW13 (Figure 1(b)) and H295R (Figure 1(c)) cells stimulated with increasing concentrations of IGF-I (1 to 50 nM) for 24 hours up to 7 days. Such a stimulatory effect is confirmed by [^3^H]thymidine uptake experiments in H295R cells (Figure 1(d)).

**Figure 2:**
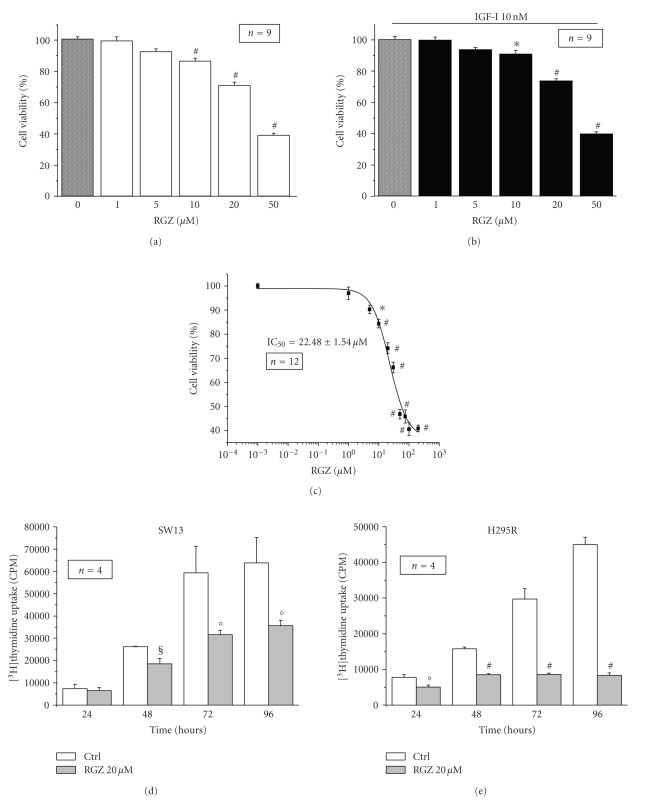
*Dose dependent effects of RGZ on adrenocortical carcinoma cell proliferation*. The dose dependent effect of RGZ on SW13 cell proliferation was evaluated by MTS assay at 48-hour stimulation in the absence (a), (c) or presence (b) of 10 nM IGF-I. A complete dose response curve for RGZ (10^−3^ to 2·10^2^ *μ*M) on cell proliferation in SW13 is reported (c), showing the IC_50_ for RGZ calculated by ALLFIT program [17]. Data represent mean ± SE percentage of OD over controls (RGZ 0 *μ*M). Statistical significance versus respective control (RGZ 0 *μ*M):*P < 0.05, #*P* < 0.001. The time dependent effect of RGZ administration (20 *μ*M) on cell proliferation was evaluated at the indicated times in SW13 (d) and H295R (e) by [^3^H]TdR incorporation. Data represent mean ± SE percentage of cpm over untreated control. Statistical significance versus respective Ctrl:°*P* < 0.01, §*P* < 0.005, #*P* < 0.001.

**Figure 3:**
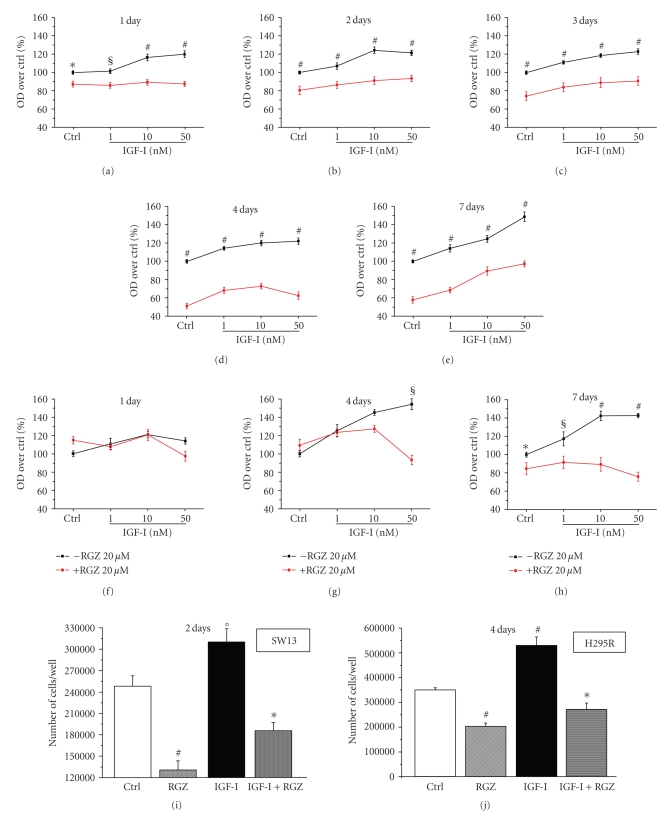
*IGF-induced cell proliferation is differently affected by RGZ in SW13 and H295R cell lines*. SW13 (a)–(e) and H295R (f)–(h) proliferation was evaluated by MTS assay following stimulation with increasing doses of IGF-I in the presence or absence of 20 *μ*M RGZ for the indicated times. Mean ± SE percentage of OD over respective control. Statistical significance:*P < 0.05, §*P* < 0.005, #*P* < 0.001 versus the corresponding points with RGZ, *n* = 6. Total number/well of SW13 (i) and H295R (j) cells treated with 10 nM IGF-I and 20 *μ*M RGZ for 2 and 4 days, respectively, was evaluated by cell counting under optical microscope. Data are expressed as mean ± SE of cell number evaluated in triplicate in three independent experiments.°*P* < .01, #*P* < .001 versus Ctrl;*P < .001 versus IGF-I.

Following 24-hour treatment, RGZ reduces cell viability in a dose-dependent manner in SW13 cells treated (Figure 2(b)) or not (Figure 2(a)) with 10 nM IGF-I, showing an IC_50_ of 22.48 ± 1.54 *μ*M (coefficient of variation 6.9%) as calculated with ALLFIT program [17], Figure 2(c). Interestingly, the IC_50_s derived from the two RGZ dose response viability curves obtained for IGF-I-treated and untreated cells are not statistically different, suggesting that the effect of RGZ is similar independently of exogenous IGF-I stimulation. Thus, we chose to use 20 *μ*M RGZ in all the experiments. The inhibitory effect of RGZ on cell proliferation in basal conditions is significantly increased with the time of incubation as evaluated by [^3^H]thymidine uptake experiments in both SW13 (Figure 2(d)) and H295R (Figure 2(e)) cells. Again, the negative effect on cell viability exerted by RGZ is similar in IGF-I treated and untreated SW13 cells (Figures 3(a)–3(e)). Conversely, in H295R cells where the effect of RGZ requires longer times to become evident, RGZ is also able to revert IGF-I-stimulation (Figures 3(f)–3(h)). Both IGF-I-stimulated proliferation and the inhibitory effect of RGZ are confirmed by SW13 (Figure 3(i)) and H295R (Figure 3(j)) cell counting at 2- and 4-day incubation, respectively.

To elucidate the intracellular mechanism by which RGZ affects adrenal cancer cell proliferation, we investigated the effects of the drug on the two main intracellular signaling pathways engaged by the activated IGF-IR, namely, the phosphatidyl inositol 3 kinase (PI3K)-Akt cascade and the extracellular signal-regulated (ERK) signaling [18].

**Figure 4:**
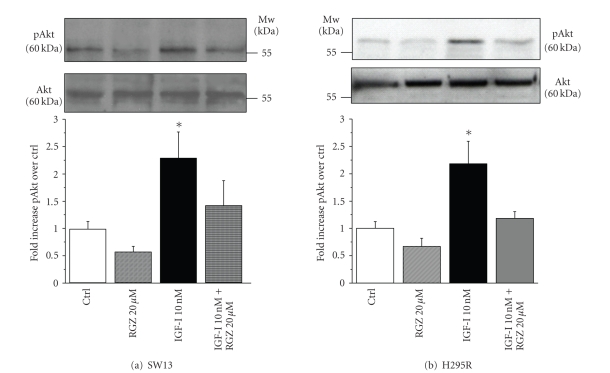
*RGZ blunts IGF-I stimulation of Akt phosphorylation/activation*. Western blot analysis of SW13 (a) and H295R (b) cell lysates following 15-minutestimulation with 10 nM IGF-I and 20 *μ*M RGZ, reveals an increased phosphorylation of Akt in Ser473 following IGF-I, which is reverted by RGZ. Lane protein normalization for Akt is shown in the middle panels. Molecular weight markers are indicated. Mean ± SE of phospho-Akt band intensity over Ctrl is shown in 4 independent experiments for SW13 (a) and H295R (b) cells (lower panels). Statistical significance:*P < .05 versus Ctrl or IGF + RGZ.

**Figure 5:**
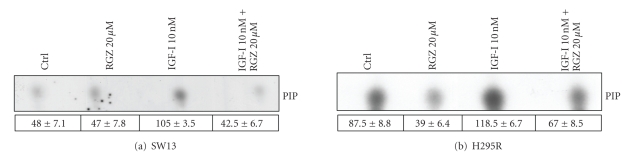
*RGZ inhibits IGF-I stimulated PI3K*. PI3K activity in SW13 (a) and H295R (b) treated or not for 15 minutes with 10 nM IGF-I and 20 *μ*M RGZ was evaluated by an in vitro assay after immunoprecipitation with an antibody against the PI3K regulatory subunit p85. The spots correspond to PI3K catalytic product [^32^P]phosphatidyl inositol phosphate (PIP). Representative of two similar experiments. Mean ± SE (*n* = 2) of phospho-Akt band intensity (arbitrary units) is shown in the table under each blot.

**Figure 6:**
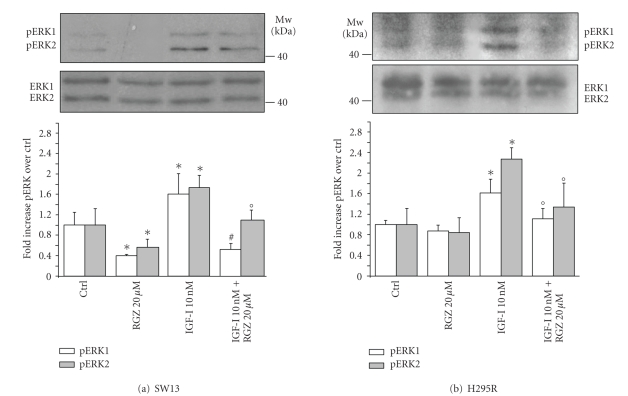
*RGZ dampens IGF-I stimulated ERK1/2 phosphorylation*. Western blot analysis of SW13 (a) and H295R (b) cell lysates following 15-minutes stimulation with 10 nM IGF-I and 20 *μ*M RGZ, reveals an increased phosphorylation of ERK1/2 following IGF-I, which is reverted by RGZ. Lane protein normalization for ERK1/2 is shown in the middle panels. Molecular weight markers are indicated. Mean ± SE of phospho-ERK1/2 band intensity over respective Ctrl is shown in 3 independent experiments for SW13 (a) and H295R (b) cells (lower panels).*P < 0.05 versus respective Ctrl;°*P* < .05 and #*P* < .005 versus IGF-I.

Rapid stimulation (15 minutes) of SW13 (Figure 4(a)) and H295R (Figure 4(b)) cells with 10 nM IGF-I determines an increased phosphorylation of Akt in Ser473, resulting in the activation of the enzyme (Figure 4, upper and lower panels). Concomitant addition of 20 *μ*M RGZ for 15 minutes in the presence or absence of IGF-I interferes with Akt phosphorylation/activation in SW13 (Figure 4(a)) and H295R (Figure 4(b)) cells. The inhibitory effect of RGZ is statistically significant versus IGF-I only, but this trend is also present on active Akt in basal conditions (Figure 4 lower panels). The in vitro immunokinase assay for PI3K performed on SW13 (Figure 5(a)) and on H295R (Figure 5(b)) lysates demonstrates a rapid activation (15 minutes) of the enzyme by IGF-I, which is reverted by co-incubation with RGZ. The inhibitory effect of RGZ is evident in basal conditions in H295R cells only (Figures 5(a), 5(b)). Similarly, RGZ interferes with IGF-I-rapid stimulation of phosphorylation/activation of ERK1/2 in SW13 (Figure 6(a)) and H295R (Figure 6(b)), but, conversely, the effect of RGZ in basal conditions is evident in SW13 cells only (Figures 6(a), 6(b)).

**Figure 7:**
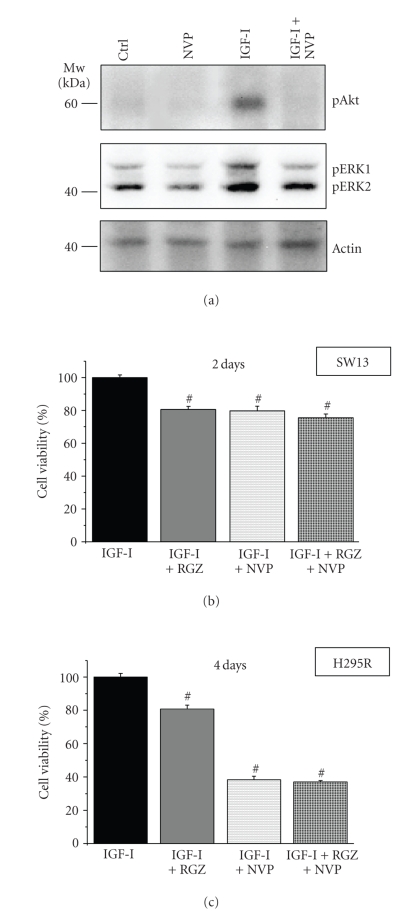
*ERK and Akt activation downstream of the IGF-IR mediates IGF-I stimulation of cell proliferation*. Western blot analysis of H295R cell lysates following 15-minute stimulation with 10 nM IGF-I in the presence or absence of 1 *μ*M NVP-AEW541 (NVP) inhibitor of the tyrosine kinase activity of IGF-IR (a). The inhibitor blocks phosphorylation of Akt (upper panel) and of ERK1/2 (middle panel) both in basal conditions and following IGF-I stimulation. Lane protein normalization for actin is shown in the lower panel. Molecular weight markers are indicated. Cell proliferation was evaluated by MTS assay following 2 or 4 day stimulation with the indicated treatments (10 nM IGF-I, 20 *μ*M RGZ, 1 *μ*M NVP) in SW13 (b) or H295R (c) cells, respectively. Mean ± SE percentage of OD over respective control IGF-I taken as 100%. #*P* < .001 versus IGF-I, *n* = 6.

In order to further demonstrate the involvement of Akt and ERK signaling downstream of the IGF-IR in mediating cell proliferation, we use the NVP-AEW541 (NVP) inhibitor of the IGF-IR tyrosine kinase activity. As shown in Figure 7(a), NVP is able to block phosphorylation of Akt (upper panel) and ERK1/2 (middle panel) both in the basal conditions and following 15-minute IGF-I stimulation. The block of the IGF-IR system by NVP results in inhibition of IGF-I-stimulated cell proliferation evaluated at 2 and 4 days of treatment in SW13 (Figure 7(b)) and H295R, respectively, (Figure 7(c)). Moreover, a further addition of RGZ to the inhibitor results in no significant reduction of cell proliferation in the presence of IGF-I compared to NVP + IGF-I (Figures 7(b), 7(c)) suggesting that RGZ growth inhibition is mediated via IGF-IR signaling.

**Figure 8:**
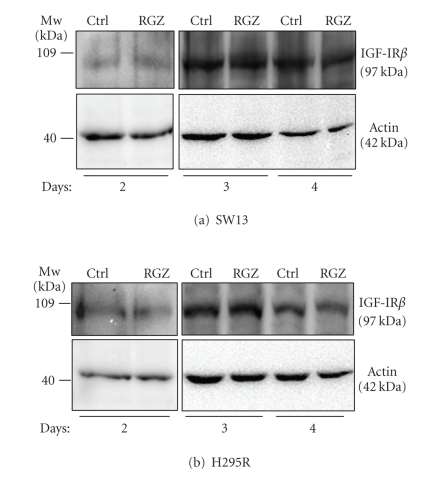
*RGZ does not affect IGF-IR levels*. Western blot analysis of SW13 (a) and H295R (b) cell lysates following 2, 3, and 4 day cell incubation in the absence (Ctrl) or in the presence of 20 *μ*M RGZ reveals no significant change in IGF-IR (upper panels). Lane protein normalization for actin is shown in the lower panels. Molecular weight markers are indicated. Representative of two independent experiments.

Finally, we investigated whether RGZ could affect not only the downstream signaling of IGF-IR, but also the levels of the receptor itself. Figure 8 shows that the IGF-IR levels do not change following up to 4-day stimulation of SW13 (a) d H295R (b) cells with 20 *μ*M RGZ.

## 4. DISCUSSION

Although adrenocortical carcinomas are very rare tumors, they are very aggressive and highly resistant to chemo- and radiotherapy. Moreover, the use of the adrenolytic agent, mitotane (o,p-DDD), is the only medical therapy available at present. Thus, a better knowledge of the molecular mechanisms underlying the tumor growth and progression is mandatory in order to develop more selective and specific treatments.

Recently, PPAR*γ* ligands have been described as suppressing tumor cell proliferation as well as inducing apoptosis and a more differentiated phenotype in several types of cancers [15], including adrenocortical carcinoma [10, 16], thus suggesting the use of these drugs as a potential new anticancer therapy. However, all these studies have been performed either in vivo, on animal models, or in vitro, on human cancer cells. Moreover, these anticancer effects have been observed with concentrations of PPAR*γ* agonists which are not only higher than the clinical doses used for T2D treatment but also affect PPAR *α* and *δ* isoforms, being no longer selective for PPAR*γ*. However, treatment with RGZ doses higher than the therapeutical 8 mg/die has been well tolerated and did not result in any increase in the percentage of adverse events compared to the placebo group in a double-blind clinical trial [19].

At present, only three papers address the effects of PPAR*γ* ligands in ACC using H295R cells as an in vitro model [10, 16, 20]. Both RGZ and pioglitazone (PIO), the most used PPAR*γ* TZD ligands, inhibit cell growth by affecting key cell cycle elements and inducing apoptosis [10, 16], also dampening cell invasiveness through reduction of metalloproteinase 2 (MMP2) expression and activity [16]. Moreover, in these cells, RGZ and PIO induce a more differentiated phenotype where steroidogenesis is increased due to a significant upregulation of MC2-R and Star expression [10].

In this paper, we use two different cell models of ACC, H295R, and SW13. H295R cells retain the ability to synthesize steroid hormones, while SW13, derived from a stage IV tumor, do not, thus suggesting them to be less differentiated than H295R. Both cell lines seem to be suitable models for studying the effects of RGZ on IGF-I/IGF-IR axis, since they express high levels of the IGF-IR, similarly to ACC tissue. In order to stimulate IGF-IR and its downstream signaling cascade, we added to the cells increasing concentrations of its elective ligand, IGF-I. Indeed, IGF-IR binds IGF-I with a 15-fold higher affinity than IGF-II [21]. However, H295R cells have been described as producing high levels of IGF-II, which acts in an autocrine-paracrine loop stimulating the IGF-IR axis even in basal conditions [8]. For this reason, in our experimental procedure, we changed cell incubation media every day to remove the endogenously produced IGF-II thus making the receptor more responsive to the exogenously added IGF-I.

In this condition, IGF-I was able to stimulate cell proliferation in a dose- and a time-dependent manner in both cell models through activation of the two main downstream intracellular signaling cascades involving phosphatidyl inositol 3-kinase (PI3K)-Akt and extracellular signal-regulated kinase (ERK1/2) [21], as described also for other tumors [18]. However, these two pathways are already active even in basal conditions, as demonstrated by the rather high levels of ERK and Akt phosphorylation as well as of PI3K activity found in the absence of IGF-I stimulation, probably due to the paracrine-autocrine effects of endogenously produced IGF-II [8].

RGZ has been demonstrated to impair IGF-I system both in vivo and in vitro by reducing IGF-I production in bone marrow cells and in the liver [22]. Moreover, troglitazone inhibits IGF-I tumor-promoting activity in mouse skin by affecting the intracellular pathway involving AMP kinase [23].

In SW13 as well as in H295R cells, RGZ was able to significantly reduce cell proliferation in a dose- and time-dependent manner as evaluated by different techniques (MTS, thymidine uptake and cell counting), with a calculated IC_50_ of 22.48 ± 1.54 *μ*M. This concentration is higher than the RGZ plasma levels estimated from the total area under the plasma concentration-time curve (Cmax 1.33 *μ*M) obtained by pharmacokinetic studies on subjects undergoing oral RGZ administration with the therapeutical dosage of 8 mg/die [24]. However, it is in perfect agreement with the doses currently used for in vitro studies, in particular in cancer cells [10, 16]. Although the RGZ inhibitory effect on cell proliferation and viability has already been reported in H295R cells [10, 16], this is the first time that RGZ IC_50_ and maximal effect have been calculated with an appropriate statistical analysis [17], thus validating the current dose of 20 *μ*M used in cancer studies and suggesting that the anti-proliferative effects of RGZ are obtained at higher concentrations than the ones effective on insulin sensitivity [13]. Toxic studies associated with a pharmacokinetic studies to define the RGZ oral dose capable of resulting in a range of 20 *μ*M circulating concentration of RGZ and to investigate the possible toxic effect associated to such doses, are mandatory in order to hypothesize a possible therapeutic use of RGZ and other TZD for ACC treatment.

In addition to its effect on cell proliferation in basal conditions, RGZ was also effective on cell growth induced by addition of increasing concentrations of IGF-I. Interestingly, such an inhibitory effect was similar in the presence or in the absence of IGF-I only in SW13 cells, while in H295R, RGZ also prevents the stimulatory effect of IGF-I. However, the inhibitory effect of RGZ was reached more slowly in H295R (4–7 days) than in SW13 (1–2 days), probably due to the differences in the kinetic of duplication between the two cell lines. Moreover, in H295R RGZ inhibition increases with the dose of IGF-I, while in SW13, the effect was similar independently of the dose of the growth factor. Consequently, the IC_50_ calculated for RGZ curve on SW13 cell proliferation was similar in the presence or absence of IGF-I.

In order to elucidate at which level RGZ interferes with the activated IGF-I axis both in basal conditions (IGF-IR activated by endogenously produced IGF-II) and following a further activation of the receptor by exogenous addition of IGF-I, we investigated the signaling pathways acting downstream of the receptor. RGZ was able to block the rapid activation of the PI3K-Akt axis induced by IGF-I. In agreement with the experiments on cell proliferation, we found that in H295R cells only, RGZ was more effective on IGF-I-treated than on untreated cells. RGZ also affected the ERK pathway through inhibition of the rapid phosphorylation-activation of ERK1 and 2 isoforms. A similar effect of RGZ on rapid phosphorylation of ERKs has been recently described in the inflammatory response of endothelial cells [14]. Such an effect is too rapid (15 minutes) to be ascribed to the classical transactivation mechanism of PPAR*γ* on specific target genes, and suggests a PPAR*γ* independent mechanism or a nongenomic activity of the receptor. Interestingly, among the anticancer action exerted by RGZ in H295R, both PPAR*γ* dependent and independent effects seem to coexist in these cells, since the PPAR*γ* antagonist GW9662 has been demonstrated to block RGZ induction of MC2R expression and cortisol secretion but not RGZ inhibition of cell proliferation (Betz et al., 2005). Further studies are required to elucidate the precise mechanism by which PPAR*γ* ligands affect rapid signaling in these cells. Interestingly, no effect of RGZ was detected up to 4 day stimulation on the level of IGF-IR in both cell lines. In accordance, Betz and colleagues described that IGF-II production increased with time despite the clear growth-suppressive effects of RGZ and PIO in H295R [10]. Our findings suggest that RGZ inhibition acts on the signaling pathways downstream but not at the level of the IGF-IR.

In conclusion, our result shed new light on the molecular mechanisms underlying cell proliferation and progression in adrenal carcinoma, contributing to demonstrate that the inhibitory effect exerted by RGZ on cell growth is due to the TDZ interferences with the two main signaling pathways downstream of the activated IGF-IR. RGZ ability to block the IGF-IR axis suggests the potential application of this molecule for the treatment of ACC.
